# Impact of malaria rapid diagnostic tests on prescription patterns of artemisinin-based combination therapy in Oyo State, Nigeria

**DOI:** 10.5281/zenodo.10878438

**Published:** 2014-02-04

**Authors:** Olusimbo K. Ige, Esther O. Ayandipo

**Affiliations:** 1 Malaria Action Program for States, Oyo Office, Ibadan, Nigeria

## Abstract

**Background:**

In the era of valuable and costly artemisinin-based combination therapy (ACT) for malaria it has been recommended that the use of ACTs is restricted to only those with confirmed positive malaria diagnosis. The potential benefits of rapid diagnostic tests (RDTs) on anti-malarial drug consumption have been demonstrated in a number of clinical trials. It is unknown if the introduction of RDTs in Nigeria has achieved the desired goal of reducing ACT consumption. This article assesses the impact of a state-wide roll-out of RDTs on ACT prescription in Oyo State, Nigeria.

**Materials and Methods:**

ACT prescribing patterns for febrile patients were compared pre- and post-RDT introduction in 106 primary health care facilities. Routine data from the national malaria control programme monthly facility summary forms were extracted for three months before and after the RDT intervention and compared using a ‘before and after’ design.

**Results:**

RDT testing rates for patients with fever revealed no trend; mean testing rate in the post RDT period was 64.5%. The mean malaria positivity rate was 71.3%, which equalled a proportional morbidity rate of 45.9% of all fever cases. ACT treatment to confirmed case ratio was consistently above the expected value of one and the ratio of treatment to tested patient exceeded one (mean ratio of 1.1) for the three months post RDT. The absolute number of ACT doses prescribed increased remarkably after the introduction of RDTs and ACTs revealing an extra utilisation of 14,199 doses, 5,534 (±517) versus 10,267 (±2,452), p<0.001. Relative Risk of ACT prescription in the post RDT period was 1.71 (1.33-2.25).

**Conclusion:**

There is notable non-adherence to RDT results, with an increase in ACT prescriptions after the initial introductory period for RDTs. This over reliance on ACTs for the management of non-malaria illness could compromise gains from reducing malaria morbidity and mortality and needs to be addressed urgently.

## 1 Introduction

Nigeria has adopted the World Health Organization’s recommendation for parasitological confirmation of malaria by rapid diagnostic tests (RDT) as one of the key interventions in improving malaria diagnosis and treatment [1,2]. As in other malaria-endemic countries, artemisinin-based combination therapy (ACT) was introduced in Nigeria as first-line anti-malaria treatment from 2005 onwards [3]. However, ACTs are relatively expensive and depends on an insecure supply of raw materials. RDTs have been recognised as crucial in ensuring targeted ACT prescription to reduce drug wastage, save money and prolong the usefulness of ACTs by reducing the emergence of resistance [4].

Although the potential benefits of RDTs on antimalarial drug consumption has been documented in some African countries [5], a number of published field trials have also revealed poor adherence to RDT results, negating the potential of RDTs to improve disease management. In Nigeria, RDTs have only recently been introduced on a large scale. Hitherto, there has only been anecdotal and unpublished health system data with regards to the impact of RDTs on anti-malarial therapy usage in Nigeria. This paper addresses the impact of the state-wide roll-out of RDTs for the management of malaria in Oyo State, south western Nigeria to guide researchers and policy makers in future deployment of RDTs.

## 2 Materials and Methods

### 2.1 Geographical area

Oyo State is the second largest state in southwest Nigeria with a land area of 27,148 km^2^ and a population in excess of 6 million. Administratively the state is divided into 33 Local Government Areas. The State operates a three-tier health care service comprising of primary, secondary and tertiary health centres spread across urban and rural areas. There are 1,648 health facilities disaggregated into 631 Primary Health Centres (PHCs), 46 Secondary Health Facilities (SHFs), 5 Tertiary Health Centres (THCs) and 968 registered private health facilities [6,7].

### 2.2 Study Design

Data from three months pre and post the RDT intervention were compared using a ‘before and after’ quasi experimental design. As part of the partner support to the Oyo State malaria control programme, 106 primary health centres out of the 631 public PHCs in Oyo State were selected based on high drug utilisation rates and availability of skilled personnel. RDTs and ACTs were distributed to the 106 selected PHCs in October 2012. RDTs had not previously been in routine use in Oyo State or used by PHC workers before October 2012. Previously, malaria diagnosis at the primary care level was predominantly based on symptoms as capacity for microscopy was minimal at this level. The RDTs distributed (SD Bioline Ag Pf) contains a glycoprotein called HRP-2 (Histidine Rich Protein 2), an antigen specific for *Plasmodium falciparum* HRP2, which has been shown to have high sensitivity and specificity in similar settings. The sensitivity, specificity, positive and negative predictive values are 100%, 98.3%, 80.0% and 100%, respectively [8].

As part of the intervention in Oyo State, one or more health care providers in each of the 106 supported PHCs was trained on RDT use as part of a 3-day malaria case management training workshop, between July and August 2012. Half a day was devoted to training participants on how to prepare and interpret results of the RDTs. Each facility was provided with RDT job-aids and interpretation guides following the training. Health workers were instructed to treat febrile patients with ACTs only if the RDT result was positive. Patients with negative RDT results were to be examined for other causes of fever or referred. The health workers were also trained on how to maintain records of RDTs used, RDT results, and ACTs dispensed and were instructed how to report these data to the local government on a monthly basis.

### 2.3 Data collection

Data were retrieved from the routine data submitted by the divided by the number of patients who received an RDT all PHCs through the local government to the State from July 2012 to December 2012. The following indicators were retrieved for the 106 supported PHCs:

- Total number of outpatients seen monthly
- Total number of fever (temperature >38.5oC) cases seen monthly
- Number of persons tested by RDT
- Number of persons positive by RDT
- Number of ACT treatments prescribed

All data were extracted from the national malaria control programme monthly facility summary forms.

### 2.4 Data analysis

Data were entered in MS Excel directly from local government summary forms. In view of the relatively short study period, trend analysis was not attempted and the data presented here are direct comparisons between weighted aver-ages for time periods (mean data for the three months pre-RDT distribution and three months post RDT distribution.)

ACT prescription data were summarised by month and weighted by the number of fever cases reported during each time period to control for fluctuations resulting from varying facility usage trends. Ratios of confirmed cases to treated cases and tested cases to treated cases are presented in a similar manner; weighting by fever patient load. Proportional malaria morbidity (PMM) was calculated as the fraction of all fever patients during a given month who were given a malaria diagnosis for the post RDT period. Test positivity rates (TPR) were calculated as the number of patients in a given month with a positive RDT result test during that same month. Ratios of ACT treatments to confirmed cases were calculated by dividing the number of ACT treatments delivered in the 3-months post RDT introduction by the number of confirmed cases (RDT positive) during that same period. This ratio equals 1 if the treatments are only delivered to confirmed cases and all suspected malaria cases are tested with RDTs. Ratios of ACT treatments to tested patients were calculated by dividing the number of ACT treatments delivered in the 3-months post RDT period by the number of patients tested during the same time period. This ratio is <1 if all suspected cases are tested and the providers deliver treatments only to RDT-positive patients. Significance testing for difference in the proportions of fever cases prescribed ACTs pre and post RDT distribution in all 106 facilities was conducted using the Chi square test. The relative risk estimate was computed for the ACT prescription rates post RDT. All statistical analyses were conducted using Stats Direct Statistical software version 2.7.9.

## 3 Results

### 3.1 Diagnosis and treatment data

Reporting data were similar for both pre and post RDT periods for the total number of facilities reporting as well as the number of months for which data were available (Table 1). A marked difference was observed in the supply and availability of ACTs in the facilities pre-RDT (5,005) and post-RDT (199,250). There were no RDTs available in any facility prior to the distribution in October, 2012. In the three months preceding RDT introduction a lower total OPD attendance was recorded (57,770) compared to the post-RDT period (62,528). However, similar proportions of all OPD patients seen were febrile in both periods (68.3%). RDT testing rates for fever patients revealed no trend with an average of 64.5% of fever patients tested over the 3 months post-RDT period. The absolute number of doses of ACTs prescribed increased significantly after the introduction of RDTs in October 2012, revealing an extra utilisation of 14,199 ACT doses (Table 1).

**Table 1.** Malaria diagnosis and treatment statistics

			Pre-RDT		Post RDT	
Total facilities reporting			106		106	
Months of data (facility month)			3 (318)		3 (318)	
Total ACTs supplied to facilities			5,005		199,250	
Total RDTs supplied to facilities			0		48,600	
Mean OPD patients seen (SD)			19,257 (3640.13)		20,843 (3263.63)	
Period	Month (2012)	Out Patient Attendance	Fever Cases (% of OPD)	RDT tests done (% of fever)	RDT Positive (% of RDT tests)	ACT pre-scribed (% of fever cases)
Pre-RDT	July	23,017	16,113 (70.0)	0	0	5,737 (35.6)
	Aug	19,003	12,545 (66.0)	0	0	5,919 (47.2)
	Sept	15,750	10,816 (68.7)	0	0	4,946 (45.7)
	**Total**	**57,770**	**39,474 (68.3)**	**0**	**0**	**16,602 (42.1)**
Post-RDT	Oct	18,826	13,568 (72.1)	6,781 (49.9)	3,988 (58.8)	8,847 (65.2)
	Nov	24,608	16,076 (65.3)	12,987 (80.8)	9,393 (72.3)	13,099 (81.5)
	Dec	19,094	13,035 (68.3)	7,759 (59.5)	6,249 (80.5)	8,855 (67.9)
	**Total**	**62,528**	**42,679 (68.3)**	**27,527 (64.5)**	**19,630 (71.3)**	**30,801 (72.2)**

### 3.2 Comparison of malaria diagnosis and treatment patterns

While the proportions of out-patients presenting with fever remained similar for the two time periods, the proportion of fever patients that received ACTs increased significantly (Table 2). Diagnosis of malaria pre-RDT was based on clinical diagnosis. It is noteworthy that the proportion of patients with clinically diagnosed malaria (as evidenced by the ACT prescription) pre-RDT was similar to the proportion of fever patients testing positive on RDT in the post-RDT period as shown by the PMM (42.1% versus 45.3%). Comparison of the proportion of fever cases prescribed ACT pre- and post-RDT revealed that in the post-RDT period there was a 71% higher chance of ACT being prescribed compared to the pre-RDT period (RR 1.71; 95% CI: 1.33-2.25).

**Table 2.** Comparison of summary measures of malaria diagnosis and treatment

Summary measure	Pre-RDT N (%)	Post-RDT N (%)	P value*
Out-patients with fever	39,474(68.3)	42,679 (68.3)	<0.001
Fever cases tested with RDT	0(0)	27,527 (64.5)	*NA
Positive RDT tests	0(0)	19,630 (71.3)	*NA
Fever cases prescribed ACT	16,602 (42.1)	30,801 (72.2)	<0.001
	Relative risk 1.71 (95% confidence interval: 1.33-2.25)		

*NA: not applicable; *: Chi Square test

### 3.3 Proportional malaria morbidity

Trends in proportional malaria morbidity among patients and test positivity rates (TPR) among those who were tested by RDTs was not apparent post-RDT. Although TPR increased over time, no corresponding increase was observed in the total recorded malaria morbidity (incidence of cases identified as malaria out of all fever cases). Whilst an average of 71.3% of tested patients were positive for malaria this constituted only 45.9% of all fever cases.

### 3.4 ACT prescription patterns

Table 3 shows the ratio of treatments to confirmed malaria cases over the 3 months post RDT period. Monthly ratios and mean ratio for this period remained higher than the expected value of 1, meaning that treatments were being administered in excess of the numbers of confirmed cases seen at the facilities. Similarly, the ratio of ACT treatment to tested patients, which was expected to be below 1, consistently exceeded 1 and a mean ratio of 1.1 was observed for the three month post-RDT period.

**Table 3.** ACT prescription patterns post-RDT introduction

	Observed	Expected
Month	Ratios of ACT treatments to confirmed cases	
*October*	8,847 : 3,988 (2.2:1)	1
*November*	13,099 : 9,393 (1.4:1)	1
*December*	8,855 : 6,249 (1.4:1)	1
Mean	10,267 : 6,543 (1.6:1)	1
Month	Ratios of ACT treatments to tested patients	
*October*	8,847 : 6,781 (1.3:1)	<1
*November*	13,099 : 12,987 (1.0:1)	<1
*December*	8,855 : 7,759 (1.1:1)	<1
Mean	10,267 : 9,176 (1.1:1)	<1

## 4 Discussion

This study evaluated artemisinin combination therapy (ACT) prescription patterns for febrile patients pre- and post-RDT implementation in selected primary health care facilities in Oyo State. These data demonstrate a promising increase in the use of RDT for malaria diagnosis after its introduction in Oyo State. Although testing rates were less than optimal with a 3 month average of 65% among fever patients, similar studies in other African studies have reported long lag periods of up to 18 months before testing rates rose above 80% [9]. What is bothersome however, is the marked increase in ACT prescriptions following RDT introduction. Although the increased availability of ACTs might be partly responsible for this increase in drug utilisation, other studies have reported reductions in ACT consumption even following increased availability of ACTs [5]. The concern here is not only the financial implication of this ACT wastage but the public health implication of misdiagnosis and inappropriate treatment of non-malaria illness. The potential increased mortality from other infections would ultimately negate the possible gains from reduction in proportional malaria mortality due to the increased mortality from other infections. This is especially important in children where mortality due to non-malarial febrile disease is exerting a toll almost as high as malaria [10].

The observed non-adherence to RDT results is a common finding in Africa [4,11,12]. The almost equal ratio of testing rates to ACT treatment lends credence to the lack of faith in test results as documented in other studies [11,13]. This may be related to the deficiency in knowledge of non-malarial fever management and the fear of doing nothing [14]. This is an indication for integrated case management training and additional clinical mentoring for PHC staff. The countries that have successfully overcome this challenge have utilised one or more of the following strategies: a) Charging a fee for first-line antimalarial drugs was thought to have contributed to adherence to diagnostic results (non-treatment of RDT-negative cases) in Senegal [9], b) a comprehensive supervisory programme, which provided regular contact with primary health care workers, c) an expanded roll-out of RDTs such that RDT use became the norm rather than being confined to certain clinics or regions [13], and d) a quality assurance system to demonstrate the sensitivity of a particular RDT product to remove fears of false-negative results [15]. In Senegal, dissemination of data to community based organisations to increase public awareness of the national malaria policy and guidelines, and the engagement of key opinion leaders to advocate RDT usage were other strategies utilised to improve testing rates and adherence to test results. These measures may also be useful in Oyo State.

The findings from this study need to be interpreted with the following considerations. The attendant data quality issues with the use of routine data is also applicable to this study such as under or over reporting of actual clinical practices. There might have been less pressure for accurate reporting of drug usage prior to the RDT and ACT introduction, which could have led to underreporting of treatments in the pre-RDT period. However, this measurement error would have biased the results toward the null hypothesis, which still indicates that RDTs had no effect on the use of antimalarial drugs. There is also a possibility that prescription practices may differ from one facility to an-other based on staff skill and experiences. This study could not directly address provider compliance with test results at an individual patient level using the dataset at hand. It is also difficult to completely attribute these results directly to the roll-out of RDTs due to the many potential confounding factors which cannot be controlled with the use of retrospective data. However, findings indicate a need for further evaluation of the influence of RDTs on prescription practices in other settings in the presence of increased availability of free antimalarials to assess consistency of these results.

## 5 Conclusions

The introduction of RDTs in Oyo State did not result in a reduction in ACT prescription compared with the pre-RDT period even with increasing RDT testing rates. A paradigm shift from clinical management of patients without evidence of malaria infection with ACTs is required. Continued monitoring of RDT rollout will also be needed to assess whether these observed case management practices would be replicated in other states. Additional measures such as closer supervision and community engagement may be needed to improve ACT prescription practices in Oyo State.
